# Factors shaping the implementation of the SAFE strategy for trachoma using the Consolidated Framework for Implementation Research: a systematic review

**DOI:** 10.1080/16549716.2019.1570646

**Published:** 2019-02-18

**Authors:** Patricia Maritim, Joseph Mumba Zulu, Choolwe Jacobs, Mumbi Chola, Gershom Chongwe, Jessy Zyambo, Hikabasa Halwindi, Charles Michelo

**Affiliations:** a Department of Health Promotion, Economics and Policy, School of Public Health, University of Zambia, Lusaka, Zambia; b Department of Epidemiology and Biostatistics, School of Public Health, University of Zambia, Lusaka, Zambia

**Keywords:** Trachoma, implementation, CFIR, determinants, SAFE strategy

## Abstract

**Background**: The SAFE strategy (surgery for trichiasis, antibiotics for active infection, facial cleanliness and environmental improvement) is the World Health Organization (WHO) recommended guideline for the elimination of blindness by trachoma by the year 2020.

**Objective**: While evaluations on the implementation of the SAFE strategy have been done, systematic reviews on the factors that have shaped implementation are lacking. This review sought to identify these factors.

**Methods**: We searched PUBMED, Google Scholar, CINAHL and Cochrane Collaboration to identify studies that had implemented SAFE interventions. The Consolidated Framework for Implementation Research (CFIR) guided development of the data extraction guide and data analysis.

**Results**: One hundred and thirty-seven studies were identified and only 10 papers fulfilled the eligibility criteria. *Characteristics of the innovation* – such as adaptation of the SAFE interventions to suit the setting and observability of positive health outcomes from pilots – increased local adoption. *Characteristics of outer setting* – which included strong multisectoral collaboration – were found to enhance implementation through the provision of resources necessary for programme activities. When community needs and resources were unaccounted for there was poor compatibility with local settings. *Characteristics of the inner setting* – such as poor staffing, high labour turnovers and lack of ongoing training – affected health workers’ implementation behaviour. Implementation climate within provider organisations was shaped by availability of resources. *Characteristics of individuals* – which included low knowledge levels – affected the acceptability of SAFE programmes; however, early adopters could be used as change agents. Finally, the use of engagement strategies tailored towards promoting community participation and stakeholder involvement during the *implementation process* facilitated adoption process.

**Conclusion**: We found CFIR to be a robust framework capable of identifying different implementation determinants in low resource settings. However, there is a need for more research on the organisational, provider and implementation process related factors for trachoma as most studies focused on the outer setting.

## Background

Trachoma is the leading infectious cause of preventable blindness and is thought to be a public health issue in 42 countries globally. It is caused by recurring infection by the bacteria *Chlamydia trachomatis*. Trachoma is characterised by multiple stages classified according to whether symptoms are associated with (i) active infection commonly observed in children – trachomatous inflammation follicular (TF) and trachomatous inflammation-intense (TI) or (ii) with corneal scarring observed in older children and adults – trachomatous scarring (TS), trachomatous trichiasis (TT) and corneal opacity (CO) [1]. The observation of the different disease stages within any given population is dependent on the duration, severity and number of infections that occur over time [2].

Almost 200 million people are thought to be living in trachoma endemic regions, with 1.9 million people suffering from blindness and visual impairment as a result of trachomatous trichiasis and resultant corneal opacities [3]. Prevalence rates are highest in poor and developing countries characterised by low levels of personal and communal hygiene as shown by infection pooling at community and household levels. This suggests that regular contact is necessary for the transmission of infection directly from eye to eye through formites, eye-seeking flies and infected ocular and nasal secretions on fingers [4].

A resolution passed by the World Health Assembly in 1998 called for the elimination of blinding trachoma by the year 2020. The World Health Organization (WHO) through the Global Alliance for the Elimination of Trachoma by the year 2020 (GET 2020) advocates for the implementation of the full SAFE strategy denoting surgery for trichiasis, antibiotic distribution, facial cleanliness and environmental improvement in countries rolling out national trachoma control programmes [5]. It is a multifaceted strategy that seeks to treat and prevent blindness caused by trachoma through (i) offering surgical treatment to individuals suffering from trachomatous trichiasis who stand highest risk of falling blind; (ii) providing antibiotic treatment as a means removing reservoirs of chlamydial infection within communities; (iii) reducing the risk of conjunctival scarring; and (iv) interrupting the transmission of infection by encouraging facial cleanliness and environmental sanitation [5,6]. The strategy targets communities encouraging their engagement through a primary healthcare approach. Full implementation of the integrated strategy has been found to be effective as different stages of the disease affect different age groups thereby requiring stage-specific interventions [2,7,8].

There has been a marked increase in the implementation of the SAFE strategy in endemic countries which can be attributed to increased political commitment towards neglected tropical disease elimination programmes and global efforts to map the burden of trachoma resulting in the availability of accurate data that guides control efforts [9]. Nevertheless, the current status of trachoma elimination shows that a large majority of endemic countries still require continuous implementation of the SAFE interventions if trachoma elimination goals are to be met [3]. Despite ongoing implementation, comprehensive systematic reviews on factors that have shaped the implementation of the SAFE strategy are lacking.

The aim of this review was to apply the Consolidated Framework for Implementation Research (CFIR) to identify factors acting as barriers and facilitators to the implementation of the SAFE strategy. The selection of CFIR was based on its ability to be applied in conducting comprehensive context assessment across multiple levels of the healthcare system. In conducting the review we identified primary research that reported on influencing factors and used CFIR’s five major domains – inner setting, outer setting, process, characteristics of the intervention and characteristics of the individuals – to analyse the implementation efforts of the SAFE strategy across multiple settings [10].

## Methods

### Search strategy

We conducted an initial scoping literature search to select the most appropriate key terms and search strategies that would meet the review’s objective. A major consideration when developing the final strategy was to make sure that it was highly sensitive to identify all existing literature on perceived barriers and facilitators to SAFE implementation. In our preliminary search we found that the assessment of trachoma interventions was sometimes conducted alongside those of other ocular diseases and perceived determinants to implementation were not always the primary outcomes of interest in studies evaluating SAFE interventions. These studies reported on factors affecting different dimensions of implementation without necessarily referring to them as acceptability, adoption, appropriateness, cost, fidelity, penetration and sustainability [11]. Furthermore, most of the captured studies measured changes in health literacy, knowledge, awareness and attitudes as outcomes of interest at implementation sites irrespective of which of the SAFE interventions had been introduced, largely because implementation of SAFE interventions is accompanied by health education and behaviour change communication. Keyword terms used were ‘Eye OR Ocular Disease AND Health Literacy’, ‘Trachoma AND Health Literacy’, ‘Trachoma Knowledge OR Awareness AND Health Literacy’, ‘Assessment AND Eye health literacy’ and ‘Assessment AND Trachoma Health Literacy’. Multiple searches were conducted in PUBMED, Google Scholar, CINAHL and Cochrane Collaboration between November 2015 and January 2017. Additional studies were identified from the reference lists of articles screened for inclusion.

### Eligibility criteria

Primary research studies that had been published in peer-reviewed journals were included in the review regardless of study design. The articles should have been published between 1998 when the SAFE strategy was adopted as the main WHO guideline for trachoma control and January 2017. The studies must have reported on perceived barriers and facilitators during any of the different phases of implementation. Facilitators and barriers were defined as any factors that promoted or hindered the implementation of any of the SAFE interventions –surgery, antibiotic administration, facial cleanliness or environmental sanitation.

The search was restricted to peer-reviewed articles published in English. Editorial and personal opinion articles were excluded. Studies that measure changes in trachoma health literacy, knowledge, awareness and attitudes but do not mention the implementation of SAFE were also excluded from the review.

### Study screening and selection

PM conducted the database searches and imported eligible studies into EndNote for reviewer access. The selection of studies was conducted using the Preferred Reporting Items for Systematics Reviews and Meta-Analyses (PRISMA) guidelines. In accordance with the guidelines from the initial 117 studies identified, 20 duplicates were excluded. PM, CJ and JMZ independently screened the titles and abstracts of the articles using the inclusion/exclusion criteria. Wherever discrepancies arose they were discussed and decisions on inclusions made jointly. All the articles that were excluded based on their titles (80) either measured health literacy in general or health literacy, knowledge, awareness and attitudes for specific ocular diseases that were out of the scope of this review such as glaucoma, diabetic retinopathy and age-related macular degeneration. The reference lists of the remaining articles were checked to identify any relevant articles that may not have arisen in the electronic search. A total of 37 abstracts were screened. Given that implementation determinants were not always the primary focus of the studies, abstracts that did not provide a lot of information but were felt to potentially meet the inclusion eligibility criteria were added to those going through a full text review. The full text review of 20 studies was focused mainly on (i) statistical analyses from quantitative findings, (ii) participant quotations in the results section and (iii) interpretive descriptions in the methods and discussion sections. Ten studies were found suitable for inclusion in the review as shown in Table 1.

**Table 1.** Summary of reasons for exclusion of studies from the review.

Stage of screening	Reason for exclusion from review
Titles	Studies were measuring general health literacy. Studies measured health literacy, knowledge, awareness and attitudes for specific ocular diseases such as glaucoma, diabetic retinopathy and age-related macular degeneration.
Abstracts	Studies measured trachoma health literacy, knowledge, awareness and attitudes but not as part of implementation of SAFE interventions.
Full text review	Studies did not report on perceived determinants to the implementation of SAFE.

### Quality assessment

The heterogeneous nature of the studies that were included in the review called for the use of the Critical Checklist for Public Health. The quality of the studies was gauged based on the validity, credibility and completeness of information presented as it related to (i) the study question; (ii) the study design, sampling, exposures, outcomes, confounders and other aspects of internal validity; (iii) interpretation and population relevance of the results; and (iv) the implications for implementation in their own population and public health practice [12]. Risk of bias assessment was also conducted using a checklist based on PRISMA guidelines to identify potential selection and measurement bias.

### Data extraction and synthesis

Guidance on the Conduct of Narrative synthesis in Systematic Reviews was used as the guiding framework for the data extraction and synthesis. The narrative synthesis approach which uses qualitative text to summarise and explore the relationships between data from qualitative and quantitative data was considered appropriate for this review due to the heterogeneity of the studies identified [13]. A good example of the application of this approach is the review by Iwelnumor et al. where it is used to study the sustainability of health interventions implemented in sub-Saharan Africa [14]. There are four stages in conducting a narrative synthesis: (i) identifying the theoretical basis on which the intervention works, (ii) conducting a preliminary synthesis through the extraction, transformation and translation of the data, (iii) exploring the relationships between and within the studies and (iv) assessing how robust the synthesis is [13]. We modified the first step of identifying a theoretical basis on which SAFE works due to the nature of our research question resulting in the use of CFIR in identifying determinants to SAFE strategy implementation. Consequently, CFIR was used as the pre-existing analytical framework enabling the extraction and organisation of data. The selection of CFIR was based on its ability to comprehensively identify a broad repertoire of multilevel determinants to SAFE implementation. Through its predefined list of constructs, CFIR would make it possible to capture facilitators/enhancers reported in the studies and group key themes within corresponding domains according to their degree of importance.

A deductive approach using CFIR as the coding framework was used during the preliminary synthesis to map the data. Extracted data included authors, year of publication, study objectives and design, characteristics of the study population, analysis, results and interpretive summaries. Codebook and memo templates with guidance on construct definition were obtained online at http://www.cfirguide.org/tools.html and used for coding and data extraction. PM, CJ and JMZ independently identified and extracted data and coded it based on the CFIR constructs they were judged to represent. Codes thought to occur in multiple domains were double coded. Comparisons on the coding was done by all the authors and wherever discrepancies arose they were discussed and final codes agreed to by consensus. Using the memo templates textual descriptions and summaries (narratives) with supporting information were recorded for each of the studies. All the authors reviewed the summary memos.

Grouping and clustering of the studies was done to allow cross literary comparisons through pattern identification across studies. Thereafter thematic analysis was used to identify the key determinants (themes) within the five CFIR domains. The process of translating the data made it possible for us to explore the differences and similarities in factors identified in the studies. The final stage of the narrative synthesis was the evaluation of the robustness of the analysis process which was done by looking at whether or not the factors identified were credible and comparable to the available implementation science literature as well their appropriateness in answering the review question.

## Results

### Study characteristics

**Figure 1.**
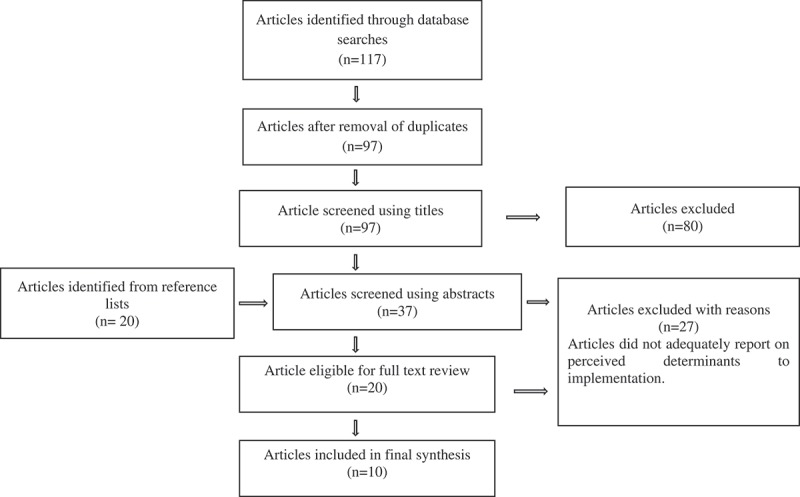
PRISMA guided selection process of studies looking at the implementation of the SAFE strategy.

Our search yielded 137 articles: 117 from the different search engines and 20 from the reference lists. PRISMA guidelines were used to identify studies that met our specific research objectives (Figure 1). Only 10 articles were eligible for inclusion in this review after the removal of duplicates and satisfaction of the eligibility criteria as described in Table 2. The countries represented by the studies were from different endemic regions globally; seven African countries, one Australian and two Asian countries. The studies were heterogeneous applying qualitative, quantitative and mixed methodologies. All the studies that had a quantitative component (n = 8) used surveys. Focus group discussion, in-depth interviews, direct observation and document review were used in the studies to obtain qualitative data (n = 4). The most common unit of analysis was the consumer level (n = 8).

**Table 2.** Summary of included studies that evaluated aspects of implementation effectiveness of the SAFE strategy.

Reference/context	Unit of analysis	SAFE component	Implementation strategies	Intervention effectiveness	Implementation outcomes	Facilitators	Barriers
1. Ajewole et al. [16] – Gambia	Consumer	S	Use of local opinion leaders – such as traditional healers and previous surgical recipients especially women for future implementation.	Poor knowledge and Awareness levels of trachoma. Perceived importance of good environmental hygiene in preventing transmission through flies. Surgical interventions considered effective but barriers inhibit utilisation. Inconsistent use antibiotics administered.	Acceptability – benefits of lid surgery were noted by recipients. Adoption – low uptake of lid surgery even when the benefits are visible. Poor compliance with antibiotic treatment.	Intervention source. Adaptability. Knowledge and belief about the intervention. Planning. Engaging.	Structural characteristics. Knowledge and belief about the intervention. Self-efficacy. Individual state of change.
2. Astle et al. [8] – Zambia	Consumer	S, A, F & E	Financial intervention – investment in the provision of water wells and training of the involved personnel. Policy – intersectoral collaboration and stakeholder engagement. Audit/feedback – screening of trachoma cases pre and post implementation. Educational outreach – ongoing educational trachoma awareness programmes.	Reduction in the prevalence of trachoma. Uptake of good hygiene practices. Surgery for trichiasis patients.	Adoption – high coverage rates for Mass Drug Administration. Implementation cost – use of a cost effective antibiotic option resulted in Appropriateness – use of local Staff familiar with the region’s cultural practices. Sustainability – local staff commitment to implementation hence continuity of interventions. Feasibility – the pilot implementation effort would inform scaling up of the SAFE strategy.	Intervention source. Evidence or observability. Trialability. Patient needs and resources. Cosmopolitanism. Readiness for implementation. Planning.	
3. Bamani et al. [19] – Mali	Consumer	F &E	Policy – implementation of a trachoma control programme through intersectoral collaboration. Educational outreach – training of the involved personnel. Mass media campaign – use of radio broadcasts to relay trachoma information.	High levels of knowledge of trachoma and positive behaviour among those who had heard the broadcast. Improved facial cleanliness.	Appropriateness – using radio messaging because a large majority of the population has access to them. Messages broadcast in local languages. Penetration – majority of the respondents had heard a trachoma message. Implementation cost – radio broadcasts were cost-effective in spreading trachoma messages.	Intervention source. Adaptability. Cosmopolitanism. Readiness for implementation. Self-efficacy. Planning. Engaging. Reflection and evaluation.	Self-efficacy.
4. Khandekar et al. [24] – Vietnam	Consumer	F	Policy – intersectoral collaboration.	Levels of knowledge and behaviour for trachoma were good.	Appropriateness – social marketing strategies and using children as the change agents of the campaign.	Intervention source. Patient needs and resources. Readiness for implementation. Planning. Engaging. Reflection and evaluation.	
5. Khandekar et al. [15] – Vietnam	Consumer	S, A, F & E	Policy – involvement of a non-governmental organisation as an implementing partner. Collaboration with different stakeholders. Financial intervention – resources set aside for provision of clean water and latrines as well as providing training. Use of local opinion leaders – women’s union, father’s union and youth union were involved. Mass media campaigns – use of marches, performances, billboards and loudspeakers to relay information. Audit/feedback – evaluation of the implementation at different points of the study. Monitoring to determine the decline of trachoma levels.	Improve knowledge and awareness of trachoma. Decline in the prevalence of Active trachoma. Improved access to sanitary latrines	Appropriateness – using organisations with social marketing skills and community commitment. Sustainability – active advocacy for good hygiene practices improved the knowledge and awareness of the targeted population over time.	Intervention source. Evidence or observability. Adaptability. Patient needs and resources. Cosmopolitanism. Implementation climate. Readiness for implementation. Planning. Engaging. Reflection and evaluation.	
6. Kuper et al. [17] – Mali, Ethiopia, Ghana, Nepal, Morocco, Niger, Tanzania and Vietnam	Organisation	S, A, F & E	Policy – intersectoral collaborations and stakeholder consultations. Audit/feedback – monitoring and evaluation of the implementation of SAFE. Audits based on a set of criteria. Reports based on findings to be used for programme planning.	Provision of antibiotics for active trachoma infection. Low number of surgeries being performed. High awareness and poor knowledge of trachoma in the programme areas.	Feasibility – insufficient coverage of antibiotic distribution programmes. Penetration – varying degrees of integration of trachoma control programmes in the healthcare system. Adoption – poor uptake of surgery for trichiasis. Fidelity – lack of consistent strategy for antibiotic distribution in the different countries.	Intervention source. Cosmopolitanism. Readiness for implementation. Planning. Reflection and evaluation.	
7. Lange et al. [18] – Australia	Provider	F & E	Policy – intersectoral collaboration.	Good awareness of trachoma. Knowledge levels of preventive measure were low. Attitudes towards improving hygiene conditions were positive. Low levels of knowledge that the region was trachoma endemic. Clinical knowledge of trachoma was also low.	Appropriateness – assessment of needs and capacities of staff in the community to optimise health promotion interventions.	Intervention source. Adaptability. Cosmopolitanism. Implementation Climate. Readiness for implementation. Self-efficacy. Planning.	Structural characteristics Implementation Climate Knowledge and belief about the intervention Self-efficacy
8. Lewallen et al. [22] – Tanzania	Consumers and Providers	F & E	Policy – intersectoral collaboration. Financial intervention – training of personnel involved. Educational outreach – training of a select number of teachers and school inspectors who were provided with communication materials in the form of teaching guides. Information dissemination – presentations made by teachers who had received the training.	Improved knowledge and awareness of trachoma. Reduction in dirty faces.	Appropriateness – health education not practical if there is inadequate access to water and latrines.	Intervention source. Adaptability. Cosmopolitanism. Readiness for implementation. Planning. Reflection and evaluation.	Patient needs and resources Implementation Climate Knowledge and belief about the intervention Self-efficacy
9. Thompson et al. [21] Guinea Bissau	Consumer	A, F & E	Policy –presence of national trachoma control policies.	Low levels of trachoma knowledge and awareness. Acceptance of the importance of hygiene and environmental cleanliness as preventive measures. Rare non-participation in mass drug administration.	Appropriateness – absence of local disease terminology and poor disease concepts thought to reduce effectiveness of health education. Future use of trained local staff. Sustainability – future use of radios to improve broadcasting of trachoma messages.	Intervention source. Self-efficacy. Planning. Reflection and evaluation.	Structural characteristics Knowledge and belief about the intervention Self-efficacy
10. Vinke et al [20] – Ethiopia	Consumer and provider	F & E	Feedback – use of the findings to inform control activities in the region.	High awareness of trachoma though knowledge of the disease was low.	Appropriateness – future programmes need to take into consideration the poverty levels, absence of water supply and proper sanitation for control efforts to be sustainable.	Intervention source. Adaptability. Readiness for implementation. Planning.	Patient needs and resources Implementation Climate Knowledge and belief about the intervention weSelf-efficacy

### Implementation outcomes

None of the studies specifically listed implementation outcomes as defined by Proctor et al. as their outcomes of interest but reported on aspects related to their measurement as shown in Table 2. Issues that arose relating to adoption included poor uptake of lid surgery for trichiasis, poor compliance with antibiotic treatment and using community engagement strategies to encourage uptake of SAFE interventions [15–17]. Steps that were taken to make the interventions more appropriate to local settings included the assessment of local conditions pre-implementation, use of local community members and local organisations during programme implementation and selection of media that would have the greatest reach [8,15,18,19]. However, where local poverty levels, absence of water supply and proper sanitation were not taken into consideration, implementation was found to have been a poor fit [20–22].

Two studies reported on varying participation rates (coverage) that are an indication of the feasibility of the implementation efforts [5,8]. Penetration was exhibited either through study respondents having heard trachoma messages (reach) or the degrees of integration of trachoma control programmes in the healthcare system [3,17,19]. Studies that sought to reduce the implementation cost did so through use of cost-effective options of antibiotics for mass distribution and media for transmission of trachoma messages [8,19]. Potential avenues of increasing the sustainability of implementation efforts reported included use of local community members who would ensure the continuity of interventions and carrying out active advocacy for good hygiene practices to improve the knowledge and awareness of the targeted population over time [8,15], as well as the use of radios to improve broadcasting of trachoma messages [21]. Acceptability and fidelity, however, were the least described outcomes [16,17].

### Implementation strategies

The different implementation strategies were categorised using the framework described by Wang et al. [23]. Various implementation strategies were reported, with policy being the most prevalent (n = 8). Policy-based strategies included presence of national trachoma control policies, intersectoral collaboration, stakeholder engagement and involvement of non-governmental organisations as implementing partners as shown in Table 2. Financial intervention was mainly through the provision of resources to provide clean water and sanitation as well as training of personnel [8,15,22]. Active implementation strategies identified include audit and feedback [8,15,17,20] and educational outreach [8,19,22]. Other strategies identified were the use of local opinion leaders, mass media campaigns and information dissemination [15,16,19,22].

The implementation determinants were grouped into the five major domains of CFIR.

### Characteristics of the intervention

#### Intervention source

The SAFE strategy is a guideline that was developed to address the problem of trachoma on a global/macro level for United Nations member states by the WHO through the Alliance for the Global Elimination of Trachoma by the year 2020. All the studies identified in this review made mention of national trachoma control programmes or other implementing bodies that had adapted the SAFE strategy for their specific contexts. Wherever development and implementation of SAFE programmes was seen as locally driven, such as through the involvement of community organisations, there were higher levels of acceptability. This could be due to an increased sense of ownership and commitment [15,18]. Utilising local community members as part of the staff or as change agents reinforced good hygiene practices because they were involved in active advocacy for behaviour change [8,15].

#### Evidence or observability

Observed positive results or stakeholders’ belief in the strength of the evidence base to justify the implementation and subsequent use of SAFE interventions were shown to affect the way certain aspects of the strategy were implemented. In Zambia the decision to use roxithromycin instead of azithromycin for mass drug administration was based on the clinical experience of one of the investigators who had been successfully using it to treat trachoma [8]. The incorporation of research findings from other settings in the formulation of implementation plans was found to improve the sustainability and continuity of the intervention [8,15].

#### Adaptability

The delivery of the SAFE strategy was done using a variety of implementation strategies. Social marketing communication approaches that took into account the unique features of the local communities were found to motivate change by making it easier to discuss changes in hygiene practices [15,16,18,20]. For instance, in Australia, among Aboriginal communities who bear the greatest burden of trachoma, discussing hygiene was considered sensitive due to existing traditional beliefs that normalise current practices and unfavourable experiences with governmental organisations which have previously appeared disrespectful to the local culture [18]. To ensure maximum reach the broadcast of trachoma health messages was done via radio because a large number of the households owned one [19].

#### Trialability

The feasibility of scaling up of the implementation of SAFE was explored in one study which provided strong evidence that the different interventions were effective in reducing the prevalence of active trachoma in a hyper endemic region. Consequently, the Government of Zambia used these findings to begin a national trachoma initiative as a preamble to the creation of a national trachoma eradication programme [8].

### Outer setting

#### Patients’ needs and resources

An understanding of the needs and resources of communities who would be the recipients of the intervention was found important in ensuring appropriate alignment of SAFE related activities. Key needs of the patients included inadequate water supply and poor sanitation. This was addressed through drilling of wells and building latrines to encourage uptake of better hygiene practices [8,15]. Introduction of trachoma health education initiatives without providing improved access to water resulted in improvement in knowledge and awareness levels but a lack of confidence in the possibility of transferring what was learnt into practice [20,22]. In communities with poor health-promoting habits the use of local opinion leaders and change agents during programme development and delivery was found to be effective in improving knowledge about the disease and improving hygiene-related behaviour [15,24].

#### Cosmopolitanism

Strong collaboration between different government agencies and non-governmental organisations was found to be important for the provision of technical and financial support to national trachoma control programmes [17]. Different ways through which support was provided included: provision of azithromycin for mass distribution by International Trachoma Initiative (ITI) [17]; development of educational curriculum guides and health promotion kits in Tanzania and Australia [18,22]; broadcasting trachoma awareness messages in Mali [19]; and improvement of water supplies, drilling wells and building latrines in Zambia and Vietnam [8,15].

### Inner setting

#### Structural characteristics

In areas where all the components of the SAFE strategy had been implemented but there was lack of continuity of care due to poorly developed healthcare systems, trachoma control was affected especially if services such as trichiasis surgery and antibiotics could only be obtained from health facilities [21]. High labour turnover and lack of ongoing clinical training resulted in staff who lacked adequate knowledge and skills for effective trachoma detection and control [18]. For instance, Lange et al. found that clinical staff and staff in schools did not know that they live in an endemic region and thought it was normal for children to have dirty faces [18]. Consultations with healthcare professionals were considered an important point of contact with community members in endemic regions, from which they could get information on how to prevent active trachoma infection [16,18,21]. Clinical staff recruited from outside local communities and who could not speak local languages were unable to effectively disseminate key trachoma health messages [21].

#### Implementation climate

Implementation climate was measured by how receptive individuals within implementing agencies were to the SAFE intervention and how the intervention activities were aligned with the routine practices of the organisation. Lack of confidence in the quality of training in trachoma control affected how able the health workers felt they were in implementing the SAFE strategy [20]. Teachers in schools where trachoma health promotion was included in the curriculum identified that inadequate inclusion of health and sanitation in the science curriculum due to time constraints or incompatibility with other curriculum priorities would affect control efforts [18,20,22]. Additionally it was felt that teaching the children about proper hygiene practices in schools that did not have adequate water supply reduced the effectiveness of what was being instructed [20,22]. Staff interviewed in different community settings in Australia before the roll-out of a health promotion kit felt that they could confidently talk about hygiene practices with others which could positively influence the kit’s implementation [18]. Provision of financial incentives encouraged adoption of good hygiene practices in Vietnamese communities [15].

#### Readiness for implementation

Financial and technical resources from non-governmental organisations and international donor organisations channelled through government agencies specifically for trachoma control programmes made it possible for successful roll-out of the SAFE strategy [8,15,17–20,22,24]. Easy access to information through educational materials and training for those involved in SAFE implementation would ensure proper service delivery. In Tanzania, school staff who were involved in the adoption of the trachoma curriculum were trained and provided with teaching guides and booklets for students [22]. Village masons in Vietnam were trained in building standard water and latrine facilities as part of facial cleanliness and environmental sanitation interventions while community trainers had coaching on how to use social marketing approaches in teaching the community about trachoma and proper hygiene practices [15].

### Characteristics of individuals

#### Knowledge and belief about the intervention

The attitudes of targeted populations to the different components of the SAFE strategy and their understanding of the underlying mechanisms through which control strategies work affected effective adoption. In some Gambian communities trachoma was not thought to be infectious and trichiasis was taken to be a symptom of other illnesses, which led to lack of skilled use of SAFE interventions [16]. The normalisation of unfavourable hygiene practices such as children having dirty faces reduced compliance with antibiotics administered, inhibited the uptake of good hygiene practices and led to the delay of treatment until the pain from trichiasis became unbearable [16,18,21]. In areas where SAFE had been fully implemented individuals were sometimes not enthusiastic about its practicality. In Ethiopia and Tanzania staff in settings where health promotion activities were in place were less enthusiastic about the how effective these interventions would be, given they were providing trachoma knowledge and SAFE related messages in the absence of adequate water supplies necessary for behaviour change [20,22]. However, some interventions’ recipients such as older women in Gambian communities who had received trichiasis surgery were mobilising younger mothers in the community to use antibiotics to prevent active infection and to receive lid surgery as they had witnessed its benefits [16].

#### Self-efficacy

Individuals who were found to be more confident in their skills and understanding of trachoma control measures are more likely to have better health-seeking behaviour and take up good hygiene practices [19]. Poor awareness of the chronic nature of trachoma in some communities made them adopt certain practices differently from the SAFE context [16]. In Gambia face washing was thought to treat the disease but it was being done among older members of the community instead of children who are the carriers of active infection [16]. In areas where health promotion activities had previously been conducted good hygiene practices were not recognised by some communities while in other communities positive behaviours such as face washing of children and proper waste disposal were exhibited [19,21]. Health worker perceptions of their ability to carry out health and sanitation education for trachoma control were influenced by their knowledge levels, skills and their confidence in the training instruction they had received [18,20,22]. In one study involving the implementation of a trachoma curriculum, teachers felt a disconnect between the theory that was taught to students and whether this could result in changes in hygiene practices given absence of adequate water supply. This led to a lack of buy-in into the implementation goals of the intervention [22].

#### Individual state of change

Implementation progress of SAFE interventions was tracked by determining an individual’s proficiency in utilising the different interventions. In one case study participants stopped applying ointment for the prevention of active inflammatory infection as soon as they felt that their symptoms had improved, increasing their chances of reinfection [16].

### Process

#### Planning

Developing a course of action to guide the implementation of SAFE activities is thought to increase implementation effectiveness. As a tool for helping to identify how the different components of the strategy would be put in place, all the studies identified in this review used epidemiological data on prevalence rates [8,15–22,24]. Wherever preliminary measures such as drilling wells and building latrines to align the needs of the involved stakeholders to SAFE interventions were taken, they facilitated uptake of trachoma health messages [7,15]. Trachoma health messages that were developed by locally based individuals were culturally appropriate and tailored to the specific contexts [18,19]. Approaches such as social marketing strategies that took into consideration the different perspectives of the stakeholders were shown to be successful in promoting good hygiene practices [15]. Tailoring the different interventions to populations that were most likely to drive infection or be effective change agents made the intervention more acceptable [16,24].

#### Engaging

Choosing the most appropriate individuals to implement and use an intervention was shown to influence how well it would be received. In the Gambia elderly women who are the custodians of community practice and recipients of lid surgery for trichiasis were found to be instrumental in mobilising young mother to take up SAFE interventions [16]. Their position as champions was enhanced by the fact that they came from the same communities and conditions as the targeted users of the interventions and thus could relate to them. Traditional leaders who also acted as opinion leaders in the community were sometimes responsible for dissuading community members from undergoing lid surgery [16]. Their inclusion in the implementation of the SAFE components could attract more people into using the interventions. Where community organisations such as women’s unions were included in the implementation of SAFE there was increased community participation [15]. The use of different media such as radio, drama performances, and billboards or during mass drug administration helped to effectively spread messages for trachoma control as well as mobilising community members [15,19,24].

#### Reflection and evaluation

Evaluating implementation efforts allows the involved stakeholders to learn from their experiences while at the same time mapping the progress they have made towards their implementation goals. In Mali and Guinea Bissau community members were found to have incomplete understanding of certain aspects of trachoma and its spread and control even after SAFE had been implemented, pointing towards the possibility that packaging and design of the health messages was not inappropriate [19,21]. The effectiveness of messages was improved when they were tailored to specific subgroups such as age bands and professions [22,24]. Social marketing coupled with the involvement of implementation champions was found to be an effective means of spreading information and encouraging good hygiene practices [15]. Increasing candidates for trichiasis surgery through outreach and active case finding could also be important in reducing national surgery backlogs [17]. Reducing the costs of conducting mass drug administration could be achieved through engagement of community directed drug distributors or the integration of trachoma drugs with other drug distribution programmes [17].

## Discussion

The SAFE strategy is a multifaceted intervention which combines multiple implementation strategies to address specific stages of trachoma [2]. The heterogeneity of the studies that were synthesised in this review provided a relatively comprehensive look at SAFE strategy implementation as they covered different levels of the healthcare system and different implementation phases. The primary focus of most of the included studies was on the effectiveness of the SAFE interventions and not on the process of implementing the interventions. Nevertheless, as the chosen analytical framework of this review CFIR proved to be a useful guide in extracting information on perceived determinants of SAFE implementation and making comparisons between findings across different studies. Construct and domain definitions found in the CFIR Wiki guide were clear and easy to apply during data extraction and coding.

The key facilitators of implementation identified included *intervention source*, *adaptability*, *cosmopolitanism*, *readiness for implementation*, *planning and engaging*. These facilitators mainly fell under characteristic of intervention, outer setting and process domains of CFIR, whereas key barriers included *structural characteristics*, *knowledge and belief about the intervention*, *self-efficacy and implementation climate* representing two domains: inner setting and characteristics of the individual. All five domains of CFIR were found to be of importance during implementation. However, of the 39 constructs some were not identified during the review, including *relative advantage*, *cost*, *culture*, *individual identification with organisation*, *networks and communication*. Most of these constructs fell within the inner setting and characteristics of individual domains. This could be due to the nature of the  studies included in the review rather than the role  these two domains play in endemic regions. Future research could explore the use of determinant frameworks such as CFIR and the Theoretical Domains Framework to explore implementation of context-specific determinants.

The use of CFIR also made it possible to see the relationship between implementation strategies, the barriers and facilitators identified and their bearing on intervention and implementation outcomes. Implementation strategies used during the implementation of SAFE in the studies varied but they were tailored to the particular settings in which they were being introduced. Policy-driven strategies enhanced the implementation process by encouraging cosmopolitanism and promoting favourable implementation climate thus making the SAFE interventions appropriate to these settings [15,17–20,22,24]. This is because trachoma elimination efforts rely on multisectoral public–private partnerships for technical support for implementation and donation of the antibiotics necessary for drug administration campaigns. Financial interventions in the form of resources set aside to build latrines, dig wells or train community members in proper hygiene practices acted as either facilitators or barriers depending on whether or not they took into account the local providers’ and patients’ needs and resources [8,15]. Adequate resources should be allocated to train and equip community members and health workers so that they are able to conduct community mobilisation, information dissemination and drug distribution boosting their self-efficacy and confidence in taking part in trachoma control efforts [25]. Use of local opinion leaders and champions was found to facilitate implementation by increasing the acceptability and sustainability of the interventions. However, this did not always lead to an increase in the level of adoption as shown by poor self-efficacy and compliance rates [15,16]. Mass media campaigns increased the reach of implementation efforts while keeping implementation costs at a minimum and ensured that local communities were more likely to remember key health messages that had been broadcast in this manner [19]. Irrespective of the implementation strategies that were used, if implementation did not take into account the inner and outer setting domains the SAFE interventions were not appropriate and sustainable.

## Conclusion

Overall, our study identified salient factors across multiple settings that are necessary for successful implementation of the SAFE strategy such as community engagement and a desire for change. However, there is a need for more literature characterising the SAFE strategy itself, organisational and provider level factors influencing implementation efforts such as leadership engagement, design of the intervention and its quality, peer pressure, external policies, incentives for implementation and the effect of networks and communication.

## Paper context

The SAFE strategy forms the basis for trachoma control efforts in endemic countries. We conducted a systematic review to identify factors affecting the implementation of the SAFE strategy using the Consolidated Framework for Implementation Research. Facilitators included use of appropriate engagement strategies, good intersectoral collaboration and adaptation of the interventions to suit local contexts. Barriers were poor implementation climate, implementing agencies’ structural characteristics, and poor knowledge and understanding of the disease and the SAFE interventions.
